# Measurement System and Testing Procedure for Characterization of the Conversion Accuracy of Voltage-to-Voltage and Voltage-to-Current Integrating Circuits for Rogowski Coils

**DOI:** 10.3390/s25206357

**Published:** 2025-10-14

**Authors:** Michal Kaczmarek

**Affiliations:** Institute of Mechatronics and Information Systems, Lodz University of Technology, 90-537 Lodz, Poland; michal.kaczmarek@p.lodz.pl

**Keywords:** harmonics conversion accuracy, signal conditioner for Rogowski coil, integrating circuit for Rogowski coil, conversion error for distorted input voltage, additional phase shift, influence of phase angle and RMS value, current-output and voltage-output active integrators

## Abstract

Rogowski coils are increasingly being used in electricity metering systems. However, owing to their operating principle, they require an additional active integrating circuit to produce an output voltage or current that is directly proportional to the input current. A signal conditioner has the most significant impact on the overall conversion accuracy of the combined transducer. In this paper, a new measurement system and testing procedure utilizing a digital power meter and arbitrary waveform generator are proposed. This approach enables the characterization of the conversion accuracy of both types of active integrators: voltage-to-voltage and voltage-to-current converters. The conversion error for distorted input voltage harmonics and additional phase shift across a range of frequencies are determined. Instead of using the actual signal from the Rogowski coil during testing —which would be challenging owing to the required high RMS value of the distorted current for its input and difficulties in accurately measuring the RMS values of harmonics and their phase angles in relation to the output voltage or current of the tested converter—an arbitrary waveform generator is used. The input voltage to the active integrating circuit replicates the output voltage of the Rogowski coil: as the harmonic order increases, its RMS voltage rises proportionally.

## 1. Introduction

Accurate current measurement and harmonics evaluation are essential in modern power systems and industrial applications because they ensure efficient operation, load monitoring, fault detection, and protection of equipment. Precise current data allow operators to detect overloads, prevent damage to generators, transformers, and motors, and maintain the stability and reliability of the electrical network. In addition, harmonics have become increasingly important to monitor owing to the widespread use of power electronics, variable-speed drives, and nonlinear loads. Harmonics can cause overheating, increased loss, misoperation of protective devices, and interference with sensitive equipment, making their measurement vital for system health and compliance with power quality standards [1,2,3,4,5,6]. Conventional inductive current transformers are widely used but can become saturated under very high currents or in the presence of DC components, limiting their accuracy under such conditions. To address these challenges, the Rogowski coil provides a non-intrusive and linear alternative for measuring high AC currents. A Rogowski coil produces a voltage proportional to the rate of change of current (di/dt). While a signal integrator is required to reconstruct the actual waveform for metering or monitoring applications, protection relays can often process the derivative signal directly, enabling fast fault detection without integration. This combination makes Rogowski coils suitable for accurate current measurement, harmonic analysis, and protection applications in modern power systems. The impact of a signal conditioner on conversion accuracy is essential in order to ensure the required measuring accuracy of the complete system. Integration can be performed digitally within a stand-alone merging unit (SAMU) instead of using an external analog integrator. By implementing the integration inside the SAMU, the system avoids the limitations of analog integrators, such as phase delays, capacitor charging effects, and sensitivity to component tolerances. Digital integration allows precise handling of low-frequency signals (e.g., 50 Hz) as well as higher frequency components and harmonics. Moreover, it enables calibration, offset compensation, and flexible filtering, improving overall conversion accuracy without relying on analog op-amps, capacitors, or resistors. In practical systems, the SAMU provides digitized measurements (sampled values) according to IEC 61850-9-2 to a phasor measurement unit (PMU), which computes voltage and current phasors, frequency, and rate of change of frequency (ROCOF) [7,8,9,10,11,12,13,14]. Digital integration inside the SAMU ensures that the signals delivered to the PMU accurately reflect both the fundamental frequency and higher order harmonics, with minimal phase distortion and conversion error. This approach simplifies the design of PMU systems, reduces reliance on analog circuitry, and allows precise digital compensation and filtering, improving the overall quality of synchrophasor measurements.

A current-output active integrator, often realized as a transconductance amplifier (Gm-amp) with a capacitor in the feedback path, is built around a differential transistor pair and biasing circuits that generate an output current proportional to the input voltage. The input stage typically consists of a differential pair of MOSFETs or BJTs, which converts the voltage difference between Vin+ and Vin− into a current. This pair is biased by a current source that sets the operating point. In many designs, this biasing keeps both transistors conducting continuously (class A operation), while in more advanced or low-power circuits, class-AB or dynamically controlled biasing may be used. The amplifier provides its output as a current, which flows into the feedback capacitor. The capacitor integrates this current, creating the voltage that closes the feedback loop and defines the integrator’s behavior. Depending on the application, the output current may also be directed into a resistive or active load, or routed to another circuit stage, but the capacitor remains the key element that turns the transconductance stage into an integrator. Local source or emitter resistors can provide degeneration, introducing negative feedback that balances the pair and improves stability, while optional driver circuits may be used to buffer or control the transistors. Global feedback, if included, further stabilizes the transconductance and sets the overall response. In operation, the signal flows from the differential input, through the biased transistor pair and current source, to the output node, where the capacitor integrates the current to produce the corresponding voltage. A voltage-output active integrator is typically implemented using an operational amplifier with a capacitor in the feedback path. The input stage consists of a differential pair of transistors that senses the voltage difference between Vin+ and Vin− and converts it into a current. This current is amplified by the op-amp’s internal gain stages and active loads to produce a voltage at the output. The feedback capacitor forces the op-amp to integrate the input signal over time, so the output voltage corresponds to the time integral of the input voltage difference. In practice, any DC offset in the input can cause the output to drift toward saturation (+Vsat or −Vsat) after some time. To prevent this, a high-value resistor is often placed in parallel with the feedback capacitor, providing a path for DC currents and stabilizing the output [15,16,17,18,19,20]. A classical voltage-output integrator exhibits high conversion accuracy at low frequencies, such as 50 Hz, because it is designed with the feedback capacitor and op-amp characteristics optimized for this frequency. At 50 Hz, the op-amp can charge the feedback capacitor effectively without introducing significant phase delay, so the RMS output voltage accurately reflects the input current of a Rogowski coil. At higher frequencies and for higher order harmonics, the voltage-output integrator becomes less accurate because the op-amp cannot charge the feedback capacitor instantaneously. The feedback capacitor and any parallel resistor directly determine the integration time constant and low-frequency behavior, which limits how quickly the output voltage can respond to rapid changes in the input current. As a result, the RMS output voltage deviates from the ideal value corresponding to the input current of a Rogowski coil, and the conversion error and phase shift for higher frequency signals and higher harmonics increase. By contrast, a current-output active integrator, using a Gm-amp with a feedback capacitor, directly forces the output current, so the current waveform accurately follows the input voltage regardless of the voltage across the capacitor. Consequently, both the phase and RMS magnitude of higher frequency signals and harmonics remain stable—the conversion error and phase shift remain low. In this type of circuit, low-order even harmonics arise mainly from output stage asymmetry, while low-order odd harmonics originate from intrinsic transistor nonlinearity. A current-output integrator is more difficult to optimize for a single desired frequency, e.g., 50 Hz, compared to a voltage-output integrator. This is because the Gm-amp stage directly forces the output current, and at low frequencies the current is very small, making it more sensitive to offsets, bias inaccuracies, and leakage currents in the circuit. As a result, the RMS value of the output current can deviate more from the ideal value corresponding to the input voltage (input current of a Rogowski Coil), leading to lower conversion accuracy at a chosen frequency, e.g., 50 Hz. By contrast, a voltage-output integrator relies on the op-amp charging the feedback capacitor, which can be accurately designed for one frequency.

The novelty of this work lies in the development of a comprehensive measurement system and testing procedure for evaluating the conversion accuracy of active integrators used with Rogowski coils. Conventionally, testing these integrators requires high-RMS distorted currents through actual Rogowski coils, making precise measurement of harmonic amplitudes and phase angles challenging. To overcome these limitations, the proposed method employs an arbitrary waveform generator to replicate the output voltage of the Rogowski coil, enabling controlled and repeatable testing conditions for both voltage-to-voltage and voltage-to-current converters. A key contribution of this study is the direct comparison of these two types of active integrators, highlighting their respective advantages and limitations in terms of conversion accuracy, phase response, and sensitivity to waveform distortions. The investigation systematically examines the influence of RMS values and phase angles of higher order harmonics on both the phase shift and conversion error of the integrators, as well as the response to purely sinusoidal signals, which serves as a baseline for performance evaluation. This provides a detailed understanding of how both distorted and ideal input waveforms affect measurement accuracy, which is critical for electricity metering and power quality monitoring applications. Furthermore, the proposed methodology offers practical benefits for researchers and engineers, as it eliminates the need for high-current setups while enabling precise characterization of harmonic effects. By quantifying conversion errors and phase deviations across a wide frequency range and harmonic content, the method supports improved design, calibration, and selection of active integrators, ultimately enhancing the reliability and accuracy of Rogowski coil-based measurement systems where analog integration is required. It is worth mentioning that the observed nonlinear effect in both tested integrating circuits/signal conditioners for the Rogowski coil is greater than that in properly designed inductive current transformers [21,22,23,24,25,26,27,28].

## 2. Measurement Setup and Evaluation Criteria for Wideband Characterization of the Conversion Accuracy of Integrating Circuits/Signal Conditioners for Rogowski Coil

**Figure 1:**
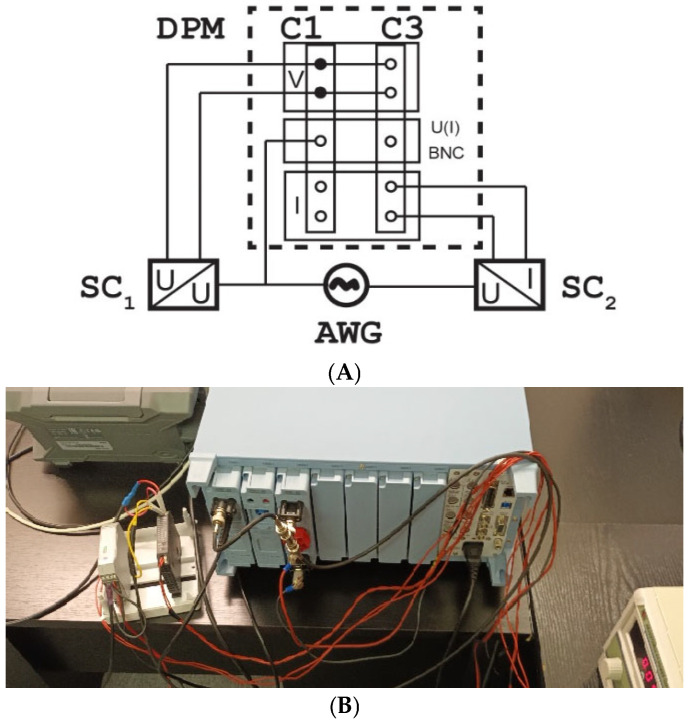
Connection diagram (**A**) and photo (**B**) of the measuring setup developed for evaluating the conversion accuracy for the harmonics of distorted input voltage.

The tested objects are two signal conditioners designed for integration of the voltage from a Rogowski coil with the current and voltage output. A connection diagram of the measuring setup developed for evaluating their conversion accuracy for the harmonics of distorted input voltage is presented in Figure 1.

The following additional notations are used in the above figure: DPM—digital power meter, AWG—two channel arbitrary waveform generator, SC1/2—1/2 signal conditioner, and U(I) BNC—current sense input of DPM.

The current sense voltage input of DPM channel 1 was applied to measure the output voltage from the combined two channels of the AWG—the input voltage for both tested signal conditioners. In order to reproduce the working conditions occurring during their operation with a Rogowski coil, the RMS value of each harmonic is multiplied by its order. The output current of the 2nd signal conditioner is measured by the current input of DPM channel 3. The voltage inputs of DPM channels 1 and 3 in this laboratory setup are used to measure the output voltage from the 1st signal conditioner. Therefore, the phase angles of the harmonics in the output current of the 2nd signal conditioner and the output voltage from the 1st signal conditioner may be determined in relation to their input voltage. The conversion error of the distorted input voltage of the *h* order harmonic is defined by the following equation for the signal conditioner with the voltage output:

(1) $$∆{CV}_{h}=\frac{{{h\cdot S}_{RC}\cdot \frac{IR_{SC}}{VOR_{SC}}\cdot U}_{hSCout}-U_{hSCin}}{U_{hSCin}}\cdot 100\%$$

where *U_hSCin_*—RMS value of the *h* order voltage harmonic measured at the input of a signal conditioner, *U_hSCout_*—RMS value of the *h* order voltage harmonic measured at the output of a signal conditioner, *h*—order of distorted voltage harmonic, *S_RC_*—sensitivity of the Rogowski coil selected or set depending on the tested signal converter (e.g., 22.5 mV/1 kA or 100 mV/1 kA), *IR_SC_*—input range of the signal conditioner (e.g., 100 A, 400 A or 5000 A RMS), and *VOR_SC_*—output voltage range of the signal conditioner (e.g., 0.225 V RMS).

The conversion error of the distorted input voltage of the *h* order harmonic is defined by the following equation for the signal conditioner with the current output:

(2) $$∆CI_{h}=\frac{{{h\cdot S}_{RC}\cdot \frac{IR_{SC}}{COR_{SC}}\cdot I}_{hSCout}-U_{hSCin}}{U_{hSCin}}\cdot 100\%$$

where *I_hSCout_*—RMS value of the *h* order current harmonic measured at the output of a signal conditioner, and *COR_SC_*—output current range of the signal conditioner (e.g., 1 A RMS).

The value of the phase shift introduced for the *h* order harmonic of the distorted input voltage by the signal conditioner is determined from the following equation:

(3) $${\delta \varphi }_{h}={-90{}^{\circ}-(\varphi }_{hSCout}-\varphi_{hSCin})$$

where *φ_hSCout_*—phase angle of the *h* order harmonic of the distorted output current/voltage of a signal conditioner in relation to the reference, and *φ_hSCin_*—phase angle of the *h* order harmonic of the distorted input voltage of a signal conditioner in relation to the reference.

During the testing procedure, a pure 50 Hz sinusoidal input voltage is applied to verify that the output contains only the fundamental component and no additional harmonics are introduced by the signal conditioner. The performance of each tested Rogowski coil signal conditioner with respect to its input voltage is characterized in terms of conversion error and phase shift under both sinusoidal and distorted conditions. For this purpose, a sinusoidal input voltage is applied across the required frequency range to evaluate the conversion accuracy of the fundamental component, followed by the application of distorted input voltages with defined harmonic content to assess the influence of waveform distortion on the conversion process and phase behavior. The behavior of different types of Rogowski coils is reflected in this procedure by varying the input voltage level of the integrator. For example, a coil with a lower sensitivity (e.g., 22.5 mV per 1 kA) is represented by a smaller RMS value of the input voltage, while a coil with a higher sensitivity (e.g., 100 mV per 1 kA) is represented by a higher RMS value of the input voltage. This approach allows for the analysis of integrator performance under conditions corresponding to a range of practical coil sensitivities, without the need for physical coils. Next, distorted input voltages with defined harmonic content are applied, including the case of a waveform composed of the fundamental frequency and a single harmonic of 5% RMS relative to the total RMS value of the distorted input voltage. For this condition, the conversion error and phase shift are determined for the harmonic component and compared with the values obtained for a pure sinusoidal input at the corresponding harmonic frequency in order to verify whether the transformation of harmonic components differs from that of single-frequency sinusoidal signals. The influence of the harmonic phase angle on the signal conditioner is examined by applying a distorted input voltage composed of the fundamental and a selected higher harmonic. The amplitude of the harmonic component is kept constant, while its phase angle relative to the fundamental is systematically varied. For each phase angle setting, the resulting conversion error and phase shift are determined, providing an evaluation of how changes in the harmonic phase angle affect the accuracy and phase response of the conditioner. Finally, the influence of the RMS value is assessed by applying sinusoidal inputs of different amplitudes and distorted inputs with varying harmonic content in order to determine how both the overall RMS magnitude and the harmonic contribution affect the conversion error and phase shift. In the case of the tested signal conditioner with current output, owing to its significantly higher conversion accuracy over a wide frequency range of the input voltage, the full application of the developed measurement procedure is necessary and therefore presented. It should be noted that, owing to significant performance variations with output load, all tests must be repeated for a given impedance. In the case of the signal conditioner with voltage output, owing to its high conversion error and phase shift, only selected results are presented to allow the comparison of the operational characteristics of both devices. As a result, the influence of nonlinear effects, even if present, may not be detected since they are obscured by low conversion accuracy even in the case of low-order higher harmonics.

**Figure 2:**
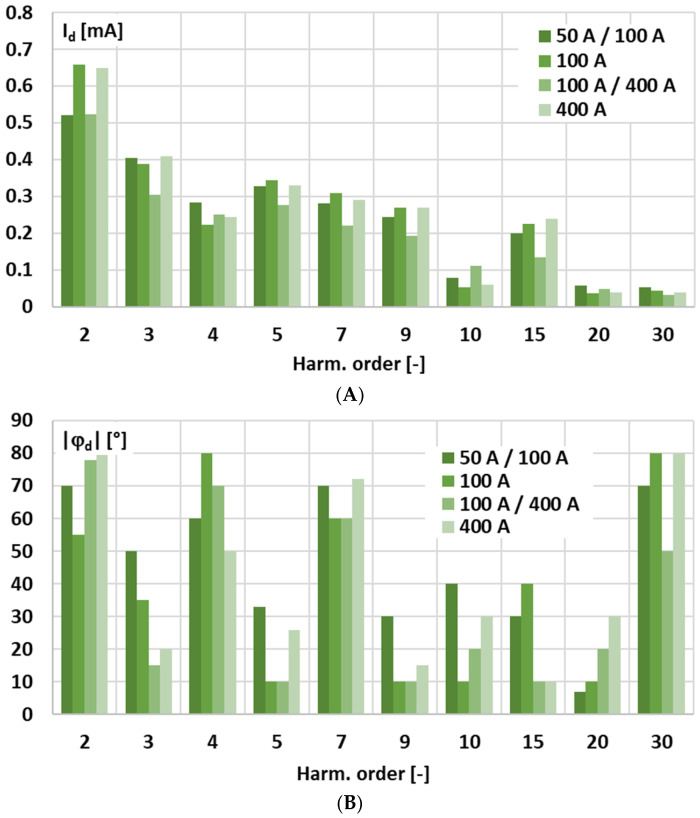
Results of the measurements obtained during the test with a 50 Hz sinusoidal input volt-age showing the RMS value (**A**) of any residual harmonic components in the current at the output of the signal conditioner and the average phase angle (**B**).

Figure 2 presents the results of the measurements obtained during the test with a 50 Hz sinusoidal input voltage, showing the RMS value (in mA) of any residual harmonic components in the current at the output of the signal conditioner and the average phase angle.

In the measurement of the output current of the tested active integrator—signal conditioner with current output—the second harmonic (Harm. order: 2) is higher than the third, reaching approximately 0.6 mA and 0.4 mA, respectively, with relatively stable averaged phases. In this type of circuit, the Gm-amp directly forces the output current, so the waveform closely follows the input voltage, which keeps both the phase and RMS magnitude stable. The higher amplitude of the 2nd harmonic compared to the 3rd harmonic is most likely caused by slight asymmetries or mismatches in the output stage or active components, such as transistors in a differential pair or elements of the current source, which tend to enhance low-order even harmonic distortion, while the intrinsic nonlinearity of the active device probably produces low-order odd harmonics. This performance will have an important influence on the determined influence of the harmonic phase angle on the conversion error and phase shift for the low-order higher harmonics of the distorted input voltage.

## 3. Conversion Error and Phase Shift Introduced by Tested Signal Conditioners for Harmonics of Distorted Input Voltage and Sinusoidal Signals

**Figure 3:**
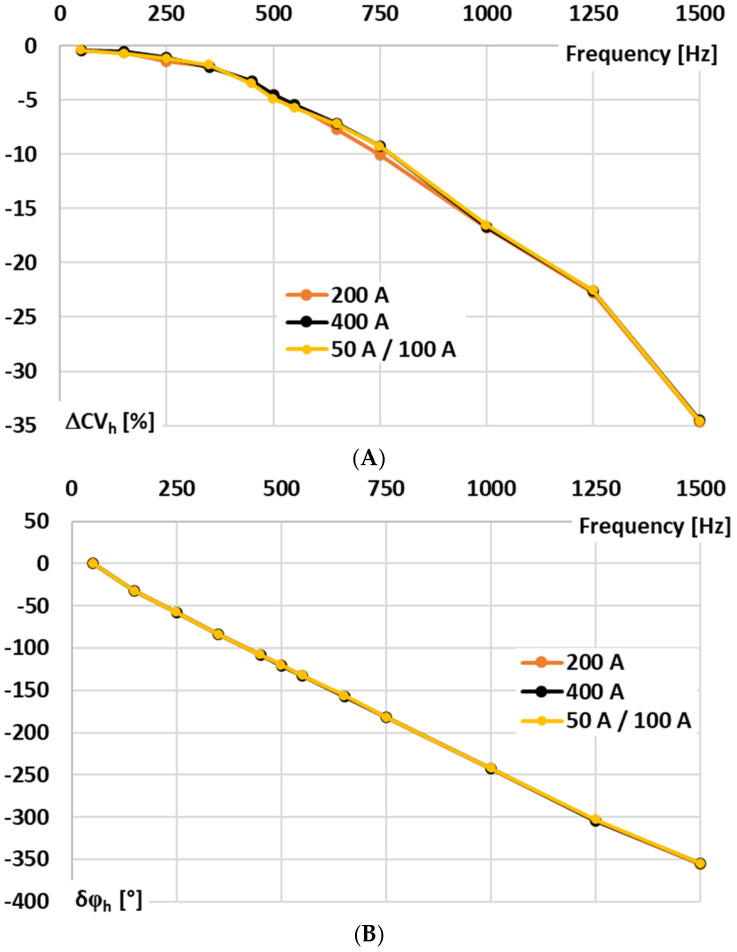
Results for the signal conditioner with voltage output: (**A**)—conversion error and (**B**)—phase shift determined for sinusoidal input voltage.

In Figure 3, the results for the signal conditioner with voltage-output are presented: A—conversion error and B—phase shift determined for sinusoidal input voltage.

In Figure 3, the following notations are used: 200 A—the results for the 200 A input range at rated input voltage, 400 A—the results for the 400 A input range at rated input voltage, and 50 A/100 A—the results for the 100 A input range at input voltage being equivalent of 50 A primary current of the Rogowski coil.

In the case of the tested signal conditioner for a Rogowski coil with voltage output, the accuracy for 50 Hz is ensured in accordance with the requirements defined in the IEC 61869-2 standard for 0.5 accuracy class inductive current transformer (conversion error −0.41, phase shift +0.22°), although this document does not concern such type of devices [29]. This good performance is retained irrespective of the RMS value of the input voltage/primary current of the Rogowski coil. However, as expected for this type of solution, the wideband conversion accuracy is limited. This issue arises because the op-amp cannot charge the feedback capacitor instantaneously. The feedback capacitor, together with any parallel resistor, defines the integration time constant and low-frequency response, thereby restricting how quickly the output voltage can follow rapid variations in the input current. Consequently, the RMS output voltage deviates from the ideal value corresponding to the Rogowski coil input current, and the conversion error as well as the phase shift increase significantly for higher frequency components and harmonics.

**Figure 4:**
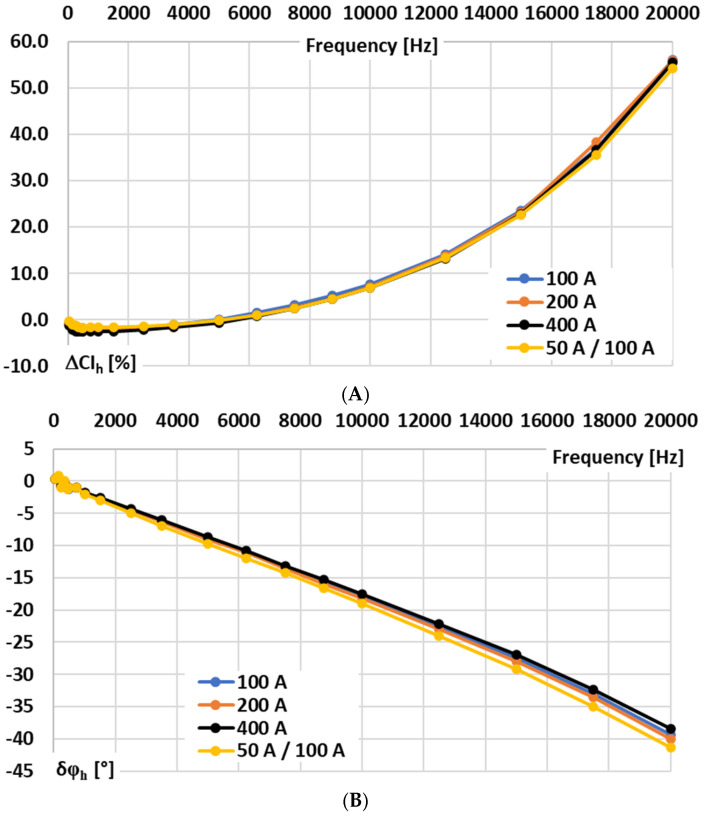
Results for the signal conditioner with current output: (**A**)—conversion error and (**B**)—phase shift determined for sinusoidal input voltage.

In Figure 4, the results for the signal conditioner with current output are presented: A—conversion error and B—phase shift determined for sinusoidal input voltage.

In the case of the tested signal conditioner for a Rogowski coil with current output, the wideband conversion accuracy is significantly higher. Therefore, the values of conversion error and phase shift were determined in the frequency range from 50 Hz to 20 kHz for sinusoidal input voltage. The results show that ±5° phase shift can be ensured up to 2 kHz, but ±5% conversion error is not exceeded up to 8 kHz. Such performance justifies the application of the developed testing procedure for the detailed characterization of its conversion accuracy and for assessing the practical influence on the wideband exploitation properties of the combined device with the Rogowski coil.

**Figure 5:**
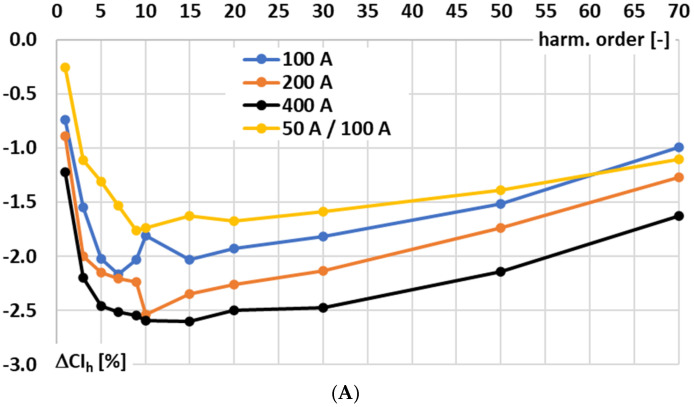

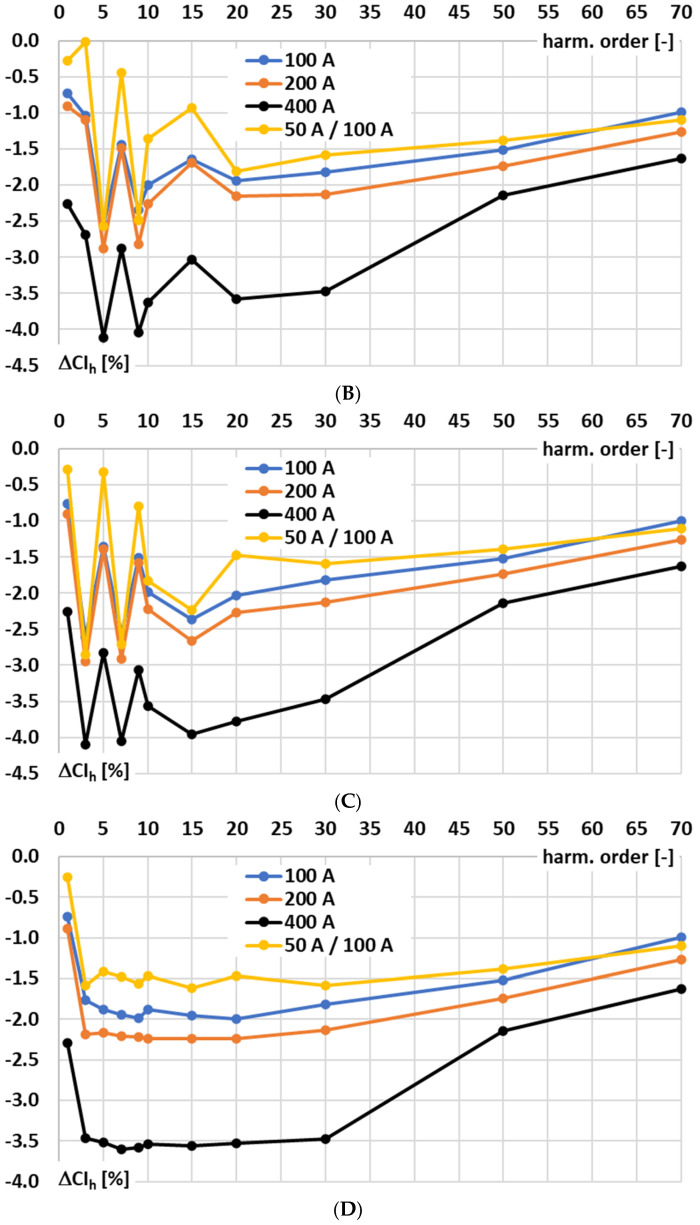
Influence of the phase angle of the higher harmonic of the distorted input voltage in relation to the main component of frequency 50 Hz on the conversion error of the signal conditioner with current output: (**A**)—0°, (**B**)—90°, (**C**)—270°, and (**D**)—180°.

In Figure 5, for the signal conditioner with current output, the influence of the phase angle of the higher harmonic of the distorted input voltage in relation to the main component of frequency 50 Hz on conversion error is analyzed: A—0°, B—90°, C—270°, and D—180°.

The results from Figure 5 confirm the influence of the phase angle of the higher harmonic of the distorted input voltage in relation to the main component of frequency 50 Hz on the conversion error of the signal conditioner with current output. This effect is caused mainly by residual harmonic components in the current at the output of the signal conditioner presented in Figure 2A. This indicates a nonlinear response of the signal conditioner at low-order higher harmonics, which was not anticipated and should be taken into account in its application. Moreover, there is a significant impact of the used current range, although during the test rated input voltage was applied. When the output current of the signal conditioner with current output is small, the circuit is particularly sensitive to amplifier offsets, biasing inaccuracies, and leakage currents. These factors lead to deviations in the RMS value of the output current from the ideal value corresponding to the input current of the Rogowski coil, thereby reducing the conversion accuracy and making it dependent on the input voltage.

**Figure 6:**
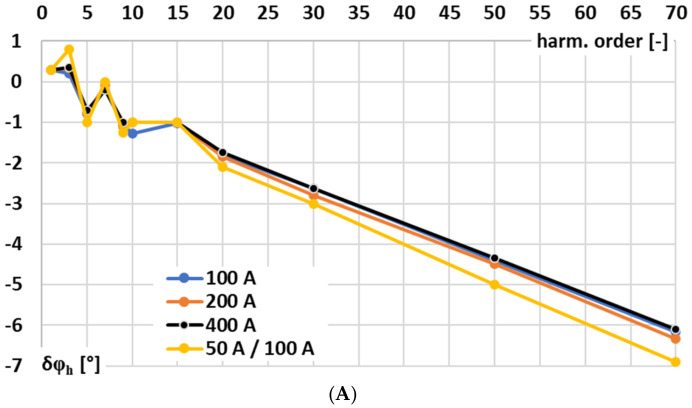

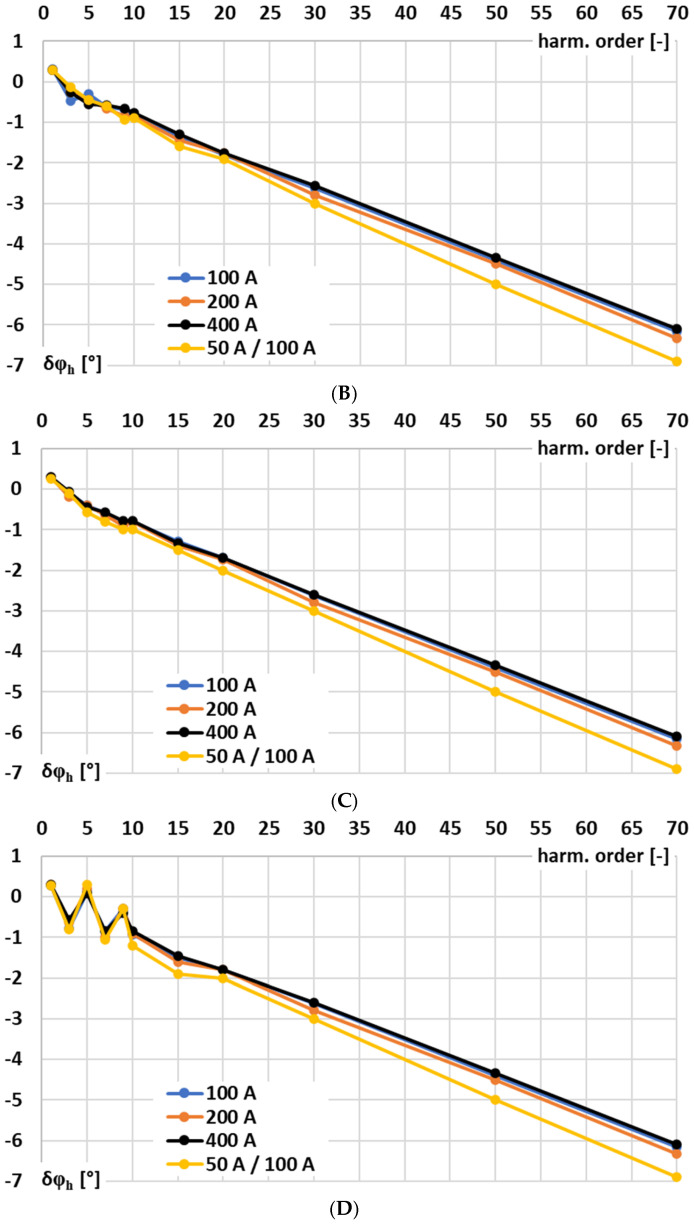
Influence of the phase angle of the higher harmonic of the distorted input voltage in relation to the main component of frequency 50 Hz on the phase shift of the signal conditioner with current output: (**A**)—0°, (**B**)—90°, (**C**)—270°, and (**D**)—180°.

In Figure 6, for the signal conditioner with current output, the influence of the phase angle of the higher harmonic of the distorted input voltage in relation to the main component of frequency 50 Hz on phase shift is analyzed: A—0°, B—90°, C—270°, and D—180°.

Figure 6 shows that the phase shift of the signal conditioner with current output for low-order higher harmonics up to the 10th harmonic is influenced by the phase angle of the higher harmonic of the distorted input voltage relative to the 50 Hz fundamental. This effect results in, as previously explained, residual harmonic components in the output current, as illustrated in Figure 2A, revealing an unexpected nonlinear response of the active integrator circuit for the Rogowski coil. There is no significant influence of the chosen input current range on the phase shift of this signal conditioner. However, some sensitivity to the lower RMS value of the input voltage is observed in the case when the distorted input voltage corresponds to 50 A RMS at 100 A RMS current range.

**Figure 7:**
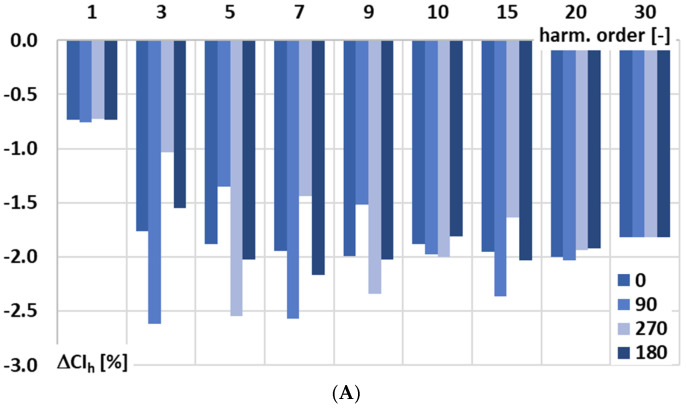

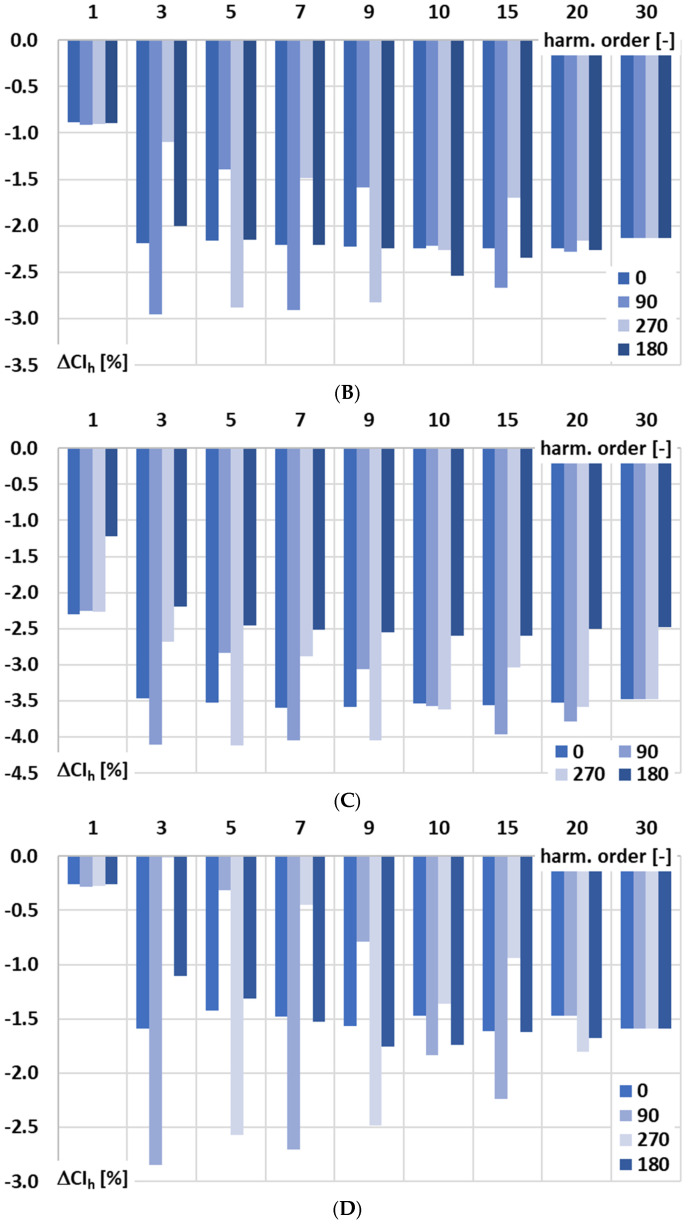
Comparison of the phase angle impact on the conversion error of the signal conditioner with current output for the harmonics of the distorted input voltage: (**A**)—100 RMS primary current (100 RMS input range), (**B**)—200 RMS (200 RMS input range), (**C**)—400 RMS (400 RMS in-put range), and (**D**)—50 RMS primary current (100 RMS input range).

Figure 7 shows a comparison of the phase angle impact on the conversion error of the signal conditioner with current output for the harmonics of the distorted input voltage under the following conditions: A—100 RMS primary current (100 RMS input range), B—200 RMS primary current (200 RMS input range), C—400 RMS primary current (400 RMS input range), and D—50 RMS primary current (100 RMS input range).

In Figure 7, the following notations are used: 0—phase angle of the higher harmonic in relation to the 1st harmonic of the distorted input voltage of the signal conditioner is 0°, 90—phase angle of the higher harmonic is 90°, 270—phase angle of the higher harmonic is 270°, phase angle of the higher harmonic in relation to the 1st harmonic of the distorted input voltage of the signal conditioner is 180°.

In the case of all low-order higher harmonics, a significant nonlinear effect is present. It subsides with an increase in their order. Taking into consideration the results presented in Figure 2A, straight cohesion occurs as in, e.g., the case of the 3rd higher harmonic. The change in conversion error for the case presented in Figure 7A is more than −1.5%. From Figure 2A, in conditions equivalent to the 100 A input range, we have about 0.4 mA, which results in such a change in the conversion error with the phase angle of the 3rd higher harmonic in relation to the 50 Hz frequency main component of the distorted input voltage for the tested signal conditioner with current output. During the test, a higher harmonic level is chosen to be 5% of the main component. Therefore, for the input voltage being equivalent to 100 A at the input range 100 A, the output current of the tested signal conditioner is 1000 mA for the main component and 50 mA for the higher harmonic. The impact of the detected residual harmonic component of frequency 150 Hz at level 0.4 mA will be ±0.8%, depending on the phase angle of the higher harmonic in relation to the main component of the distorted input voltage. The detected change was expected to be higher, as this was only one condition considered that affected the conversion accuracy at low-order higher harmonics of the tested signal conditioner with current output. In the test scenario presented in Figure 7C, for the main component of the distorted input voltage, a difference in conversion error of about −1% was detected for the case when the higher harmonic’s phase angle was 180°. This was not a coincidence—this was a thermal effect resulting in a different conversion error due to the warm-up time of the signal conditioner (5 min instead 20). According to Figure 7A–D, the value of the conversion error for the main component of the distorted input voltage ranges from −0.2% to −2.3%. While in the case of low-order higher harmonics, for the 3rd harmonic, it ranges from 0% to over −4%, and for the 5th harmonic, it ranges from about −0.2% to also over −4%, with dependence on the higher harmonic phase angle and required input voltage range. The nonlinear effect diminishes with an increase in the frequency of higher harmonic and, in the case of the 30th harmonic, the value of the conversion error varies only in Figure 7C due to the warming time influence. The increase in input range—the case presented in Figure 7C (400 A) in relation to lower ranges (Figure 7A—100 A and Figure 7B—200 A)—causes an increase in the conversion error. This may be caused by the multi-tap voltage transformer used for extension of the input range required in order to maintain a consistent voltage level for the gm-amp. Comparing the results for scenario A and D for the same input range but a reduced input value in case D, it can be observed that the conversion error also changes depending on the phase angle of the higher harmonic of the input voltage of the tested signal conditioner. Nonlinear effects are present across both the main and low-order higher harmonics of the input signal. Increasing the input range amplifies the conversion error, while phase angle variations continue to affect both main and harmonic components.

**Figure 8:**
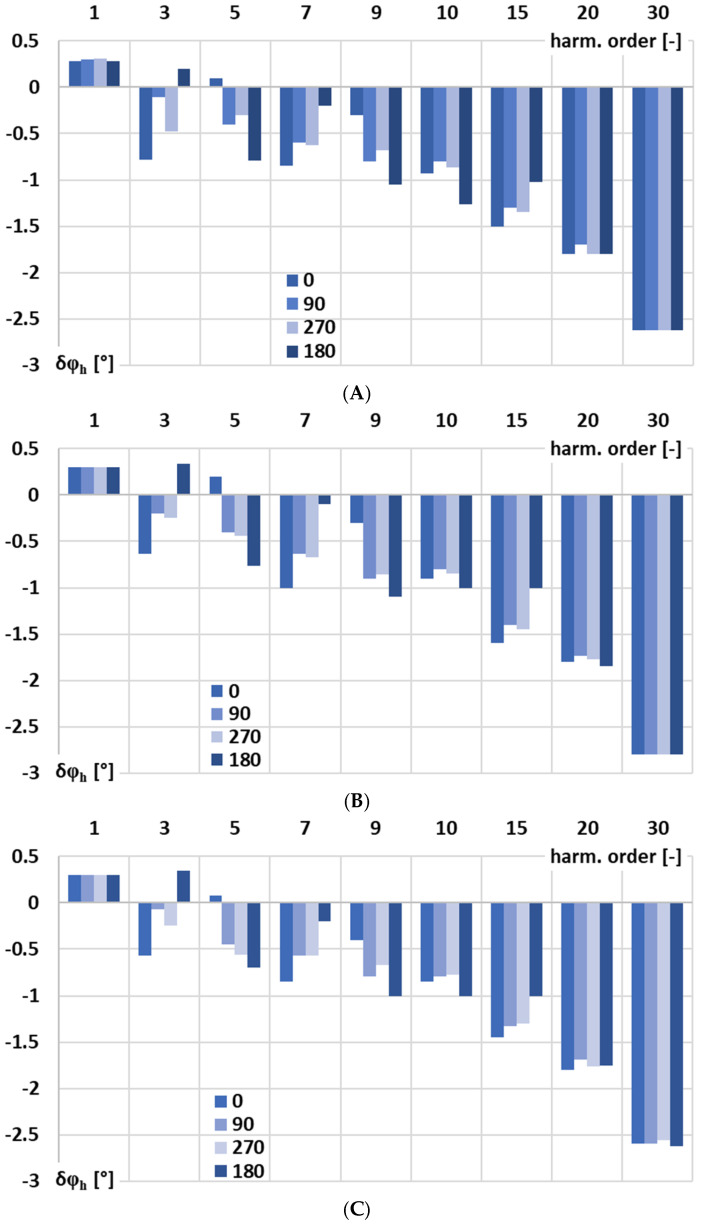

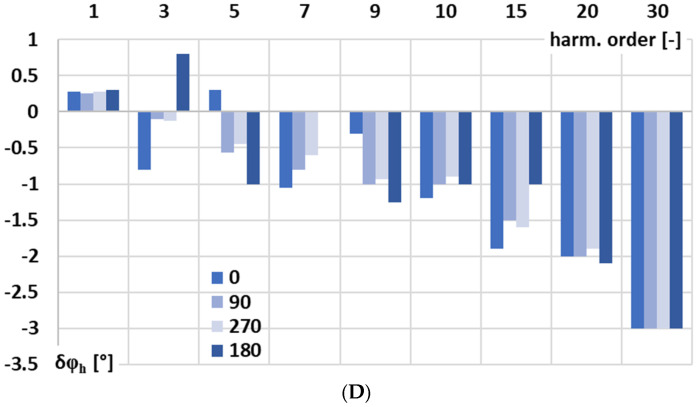
Comparison of the phase angle impact on the phase shift of the signal conditioner with current output for the harmonics of the distorted input voltage: (**A**)—100 RMS primary current (100 RMS input range), (**B**)—200 RMS (200 RMS input range), (**C**)—400 RMS (400 RMS input range), and (**D**)—50 RMS primary current (100 RMS input range).

Figure 8 shows a comparison of the phase angle impact on the phase shift of the signal conditioner with current output for the harmonics of the distorted input voltage under the following conditions: A—100 RMS primary current (100 RMS input range), B—200 RMS primary current (200 RMS input range), C—400 RMS primary current (400 RMS input range), and D—50 RMS primary current (100 RMS input range).

In all of the test scenarios presented in Figure 8A–D, for the main component of the distorted input voltage, only a small deviation in phase shift value was observed, remaining stable at around +0.3°. While in the case of low-order higher harmonics, for the 3rd harmonic, it ranges from −0.75° to +0.75°, and for the 5th harmonic, it ranges from about −1° to also over +0.25°, with dependence on the higher harmonic phase angle but with minor influence of the required input voltage range. In the case of the phase shift, the nonlinear effect is weaker than that caused by the conversion error. It is observed only in the low-order higher harmonics of the input voltage and diminishes as the harmonic frequency increases.

**Figure 9:**
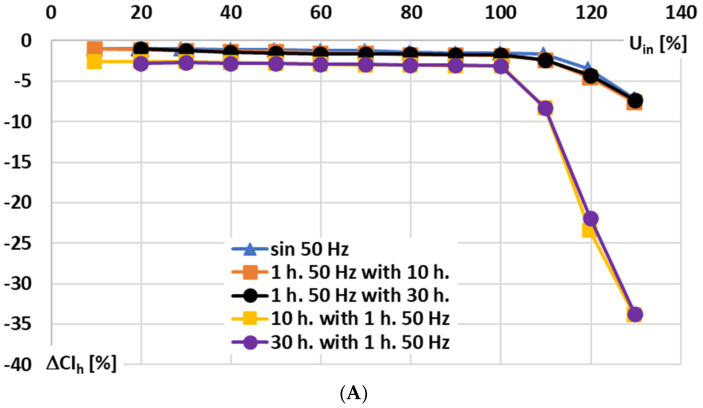

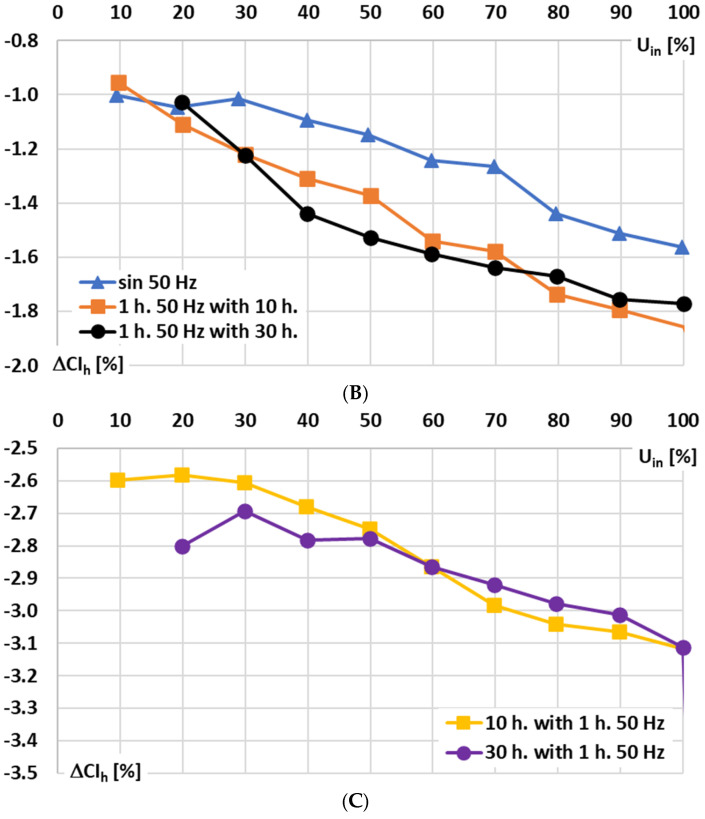
Influence of RMS values of the sinusoidal and main harmonics of the distorted input voltage on the conversion error of the signal conditioner with current output: (**A**)—10% to 140% of rated range, (**B**)—10% to 100%, and (**C**)—10% to 100% of rated range focused on the 10th and 30th higher harmonics.

In Figure 9, for the signal conditioner with current output, the influence of the RMS values of the sinusoidal and main harmonics of the distorted input voltage on the conversion error is analyzed for the following conditions: A—10% to 140% of rated range, B—10% to 100% of rated range, and C—10% to 100% of rated range focused on changes concerning the 10th and 30th higher harmonics (retained constant value 10% of the main component).

In Figure 9, the following notations are used: sin 50 Hz—sinusoidal input voltage of frequency 50 Hz; 1 h. 50 Hz with 10 h—results for 1st harmonic of distorted input voltage of main frequency 50 Hz with 10th higher harmonic; 1 h. 50 Hz with 30 h—results for 1st harmonic of distorted input voltage of main frequency 50 Hz with 30th higher harmonic; 10 h. with 1 h. 50 Hz—results for 10th harmonic of distorted input voltage of main frequency 50 Hz; and 30 h. with 1 h. 50 Hz—results for 30th harmonic of distorted input voltage of main frequency 50 Hz.

Based on the presented results, it can be seen that the rated input voltage may not be exceeded by the input voltage (Figure 9A). Only a slight difference is noticeable between the conversion error of a sinusoidal input voltage and that of a distorted input voltage harmonic of the same frequency (Figure 9B). Furthermore, an increase in the main component of the distorted input voltage leads to a higher conversion error of the higher harmonic (Figure 9C), even when its RMS value remains unchanged (the input voltage of the higher harmonic corresponds to an input current of 10 A in the Rogowski coil).

**Figure 10:**
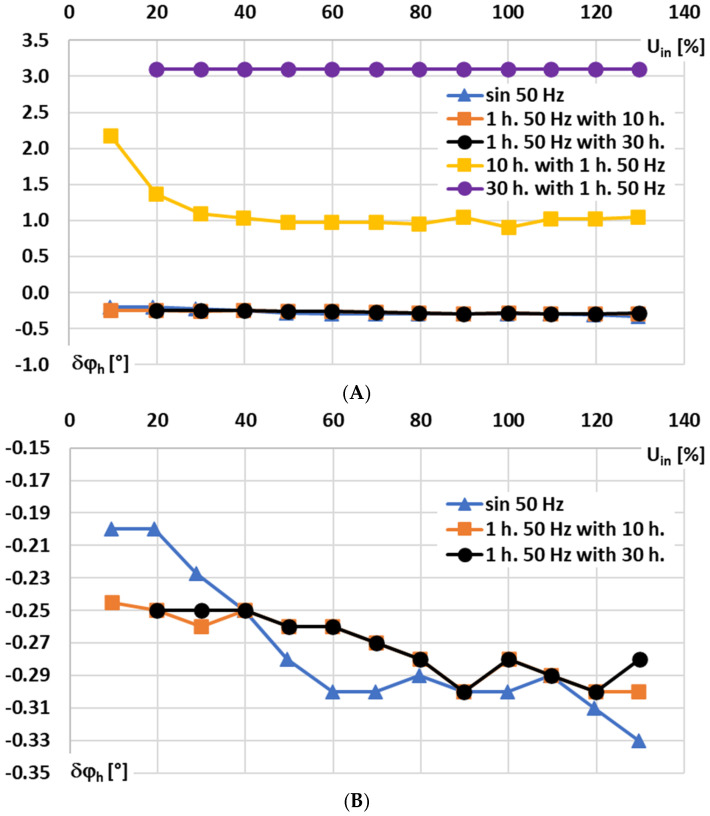
Influence of RMS values of the sinusoidal and main harmonics of the distorted input voltage on the phase shift of the signal conditioner with current output: (**A**)—10% to 140% of rated range, and (**B**)—10% to 140% of rated range focused on main 50 Hz harmonic component and sinusoidal 50 Hz.

In Figure 10, for the signal conditioner with current output, the influence of the RMS values of the sinusoidal and main harmonics of the distorted input voltage on the phase shift is analyzed for the following conditions: A—10% to 140% of rated range, and B—10% to 140% of rated range focused on main 50 Hz harmonic component and sinusoidal input voltage of frequency 50 Hz.

The results in Figure 10A,B show that the RMS value of the input voltage has no significant effect on the phase shift introduced by the signal integrator for Rogoski coils with current output for the sinusoidal or higher order harmonics of the distorted voltage. Only in the case of low-order higher harmonics, e.g., the 10th harmonic, is the RMS value of the 1st component below 30% of the rated input voltage, indicating that such an effect exists.

## 4. Conclusions

Accurate current measurement and harmonic evaluation are vital for maintaining efficiency, reliability, and power quality in modern electrical systems. Rogowski coils provide a non-saturating, wideband alternative to conventional current transformers, but their performance depends heavily on the design of the signal conditioner. The conducted evaluation demonstrates that analog active integrators, whether voltage- or current-output, exhibit significant limitations. Voltage-output integrators ensure the same performance for 50 Hz sinusoidal current as 0.5 accuracy class inductive current transformers and at the fundamental frequency (50 Hz) of distorted input voltage, but they fail to deliver reliable wideband performance due to op-amp and feedback capacitor constraints. Current-output integrators extend the bandwidth and reduce the phase error, yet they introduce considerable nonlinear effects at low-order harmonics and show sensitivity to input range and offsets. These nonlinearities are high enough to compromise measurement fidelity for distorted currents. Therefore, analog integration is unsuitable for high-accuracy harmonic monitoring and synchrophasor applications. Digital integration implemented inside, e.g., a stand-alone merging unit (SAMU), emerges as the available solution. By avoiding the limitations of analog circuitry, SAMUs are expected to enable precise calibration, offset compensation, and robust wideband accuracy, ensuring reliable Rogowski coil-based measurement systems that meet the demands of IEC 61850-compliant power networks.

This study introduced a comprehensive testing methodology for active integrators, enabling precise evaluation of conversion accuracy, phase shift, and nonlinear effects under both sinusoidal and distorted input conditions without the need for high-current setups. The results confirmed that voltage-output integrators can achieve sufficient accuracy at the fundamental frequency (50 Hz), in line with IEC 61869-2 requirements (conversion error below ±0.5% with phase shift below ±0.5°), but their performance rapidly deteriorated at higher frequencies and harmonics owing to limitations in the op-amp–capacitor integration stage, maintaining the conversion error below ±5% only up to 500 Hz but with an unacceptable 120° phase shift. By contrast, current-output active integrators based on transconductance amplifiers demonstrated significantly higher wideband conversion accuracy, maintaining the conversion error below ±5% up to 8 kHz and phase shift within ±5° up to 2 kHz. However, these circuits exhibited sensitivity to offsets, biasing inaccuracies, and leakage currents at low RMS values, which reduced accuracy for the fundamental frequency, with conversion errors of ±1% and ±3% at the higher input range and a phase shift below ±0.5°. Additionally, residual nonlinearities were observed in low-order harmonics, with the conversion error strongly influenced by harmonic phase angle and input range, although the effect diminished with increasing harmonic order. The findings emphasize that while voltage-output integrators remain suitable for applications requiring higher accuracy at 50 Hz, current-output integrators are better suited for wideband applications. The developed testing procedure proved essential in identifying nonlinear effects and quantifying their influence, offering a valuable framework for integrator design, calibration, and selection in Rogowski coil-based measurement systems.

## Data Availability

Data are provided within the manuscript.
